# PEI-Reinforced GO/g-C_3_N_4_ Composite Membrane for Salt Separation

**DOI:** 10.1021/acsomega.5c00135

**Published:** 2025-04-16

**Authors:** Wenbiao Zheng, Mingfeng Yu, Sujuan Yang, Licong Meng, Yonghe Xiu, Yifan Liu, Hanhui Lei, Terence Xiaoteng Liu, Zhanhui Yuan, Liwei Wang

**Affiliations:** † College of Environmental and Safety Engineering, Fuzhou University, Fuzhou 350108, China; ‡ College of Materials and Chemical Engineering, Minjiang University, Fuzhou 350108, China; § Department of Mechanical and Construction Engineering, Northumbria University, Newcastle-upon-Tyne NE1 8ST, U.K.; ∥ College of Materials Engineering, Fujian Agriculture and Forestry University, Fuzhou 350002, China

## Abstract

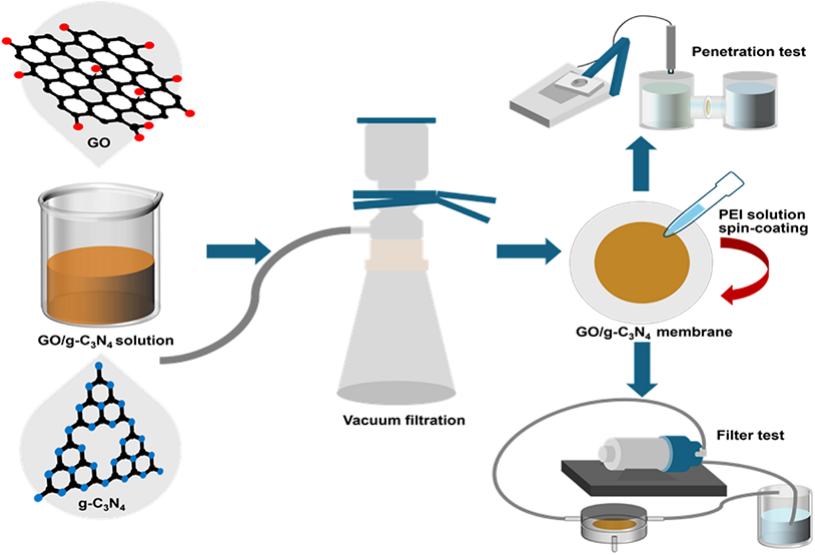

Membrane separation is an important separation and purification technology, membrane materials are the key to membrane separation technology, and the rapid development of two-dimensional materials in recent years has brought new opportunities for membrane separation technology. Graphene oxide (GO) is a hydrophilic two-dimensional material, and its unique physical and chemical structure and properties give it the potential to become a high-performance separation membrane; however, the instability limits its application. In this study, a two-dimensional material, graphite-phase carbon nitride (g-C_3_N_4_), was integrated into graphene oxide (GO) to address the challenges associated with its practical applications. This modification not only endows the material with more advantageous properties as a membrane but also introduces a heterogeneous structure that bolsters the composite membrane’s stability. This structure also enhances the permeability and selectivity of the membrane, leading to impressive results in self-driven permeation tests and staggered-flow filtration tests under applied pressure. Following additional surface charge modifications, the composite membrane demonstrated a separation efficiency of approximately 90.4% in permeation tests and an impressive separation efficiency of 63.8% in filtration tests for binary mixed salt solutions. These results indicate a significant capability for mono- and divalent ion separation, suggesting promising potential for salt separation applications.

## 1 Introduction

Nowadays, the whole world is facing the reality of high energy consumption, high pollution, and high material consumption in the chemical industry, and the problems of scarcity of freshwater resources and shortage of energy are becoming more and more serious, while the recycling of water resources is crucial to the sustainability of modern society.^1^ Therefore, the development of new and efficient water separation and purification technology is one of the major needs of the country, and membrane separation technology stands out among many separation technologies because of its energy-saving and high-efficiency advantages.^2,3^ Research on monovalent and divalent ion separation strategies based on membrane separation technology has shown great development potential for solving industrial applications such as lithium extraction from salt lakes,^4,5^ seawater desalination,^6^ and brine refining,^7^ and the demand for its application in the fields of seawater desalination, storage and conversion of energy sources, and environmental treatment has been elevated.^8−10^ However, traditional separation membranes are not able to perform efficient selective screening of mono/divalent ions with different subnanometer sizes, so further development of membrane separation technology and production of efficient ion-selective separation membranes are of great importance in the field of separation and purification.^11,12^

Membrane separation technology can be used to realize different functional applications by adjusting the physical and chemical properties of membranes.^13^ Currently, commercial ion-selective membranes are mainly traditional polymer membranes,^14^ but they are limited by the trade-off effect.^15^ It is difficult to change the interlayer environment of membranes on the subnanometer scale to achieve high flux and high ion selectivity at the same time, and in order to break through the bottleneck of this effect, new two-dimensional material membranes have become a recent research hotspot in recent years.^16,17^

Two-dimensional material membranes are assembled by stacking monolayers of 2D nanosheet materials. 2D nanosheet materials can be classified into two categories according to their structural features, one is nanosheets without regular pores on the lamellar structure, such as graphene,^18^ graphene oxide (GO),^19,20^ MXenes,^21^ and transition metal dichalcogenides (TMDC).^22^ The other is nanosheets with intrinsic micropores on the lamellar structure nanosheets, such as graphite-phase carbon nitride (g-C_3_N_4_),^23^ two-dimensional metal–organic frameworks (2D MOFs),^24^ and two-dimensional covalent organic frameworks (2D COFs).^25^ Among them, GO is a graphite derivative, which consists of sp^2^ hybridized carbon atoms forming a two-dimensional hexagonal structure similar to honeycomb with other atoms through σ bonds, and the layer is enriched with a large number of oxygen-containing groups, which has the advantages of large specific surface area, strong hydrophilicity, and easily adjustable physicochemical properties, making it an ideal membrane material.^19,26,27^ However, GO membranes are prone to swelling in water, resulting in the loss of separation performance due to the increase in interlayer spacing, making it difficult to operate effectively for long periods of time. In this regard, the introduction of cross-linking substances into the layers of GO membranes to connect adjacent nanosheets through covalent or noncovalent interactions has been widely used to control the nanochannels of GO membranes.^28,29^ For example, Wang et al.^30^ proposed a cationic amphiphilic molecule embedded into amine-cross-linked GO membranes to alter the charge environment of the nanochannels and reduce the friction between the transported substances and the channels while improving permeability and selectivity. Li et al.^31^ were inspired by biological ion channels based on ion-controlled interlayer spacing and used hydrophilic ligands to immobilize metal cations by coordination while adjusting the interlayer spacing, while the π–π and hydrogen bonding interactions between the complexes and GO enabled good retention in the interlayer to reduce the performance degradation caused by the loss of metal cations under the influence of pressure and permeate. The hydrophilic groups of the ligands simultaneously modified the chemical environment of the interlayer channels and promoted the permeability of monovalent ions.However, it is difficult to predict the deformation of nanochannels due to the insertion of special molecules and ions and the secondary contamination caused by the loss of the ions during use. The change of interlayer functional groups also brought unpredictable effects on the ion transport characteristics of the GO membranes themselves, so that the formation of heterogeneous channels through the hybrid matrix membranes has received attention from researchers.

Different from the less stable flexible 2D material GO, g-C_3_N_4_ is a rigid and stable 2D material with a graphite-like large π-bond-conjugated system of two structural units of the triazine or tri-*s*-triazine structure composed of sp^2^ hybridized carbon and nitrogen atoms.^23,32^ The aromatic heterocyclic structure of g-C_3_N_4_ provides excellent thermal stability, the intrinsic nanopores formed by the structure can provide additional transport channels for substances, and its strong interlayer van der Waals forces can remain stable in most solvents, showing excellent photocatalytic and electronic properties in the fields of water treatment, biodegradation, and semiconductors, which makes it an ideal membrane material.^33,34^ However, the stabilized g-C_3_N_4_ also has drawbacks, including small specific surface area, difficulty in exfoliation into single/few-layer nanosheets, difficulty in homogeneous dispersion in solvents, and other difficulties in practical applications, and it is usually necessary to improve the yield and dispersion of the nanosheets by means of precursor-doped elements,^35^ assisted exfoliation,^36^ and other methods.

In this study, the characteristics of g-C_3_N_4_ were enhanced by incorporating GO into the melamine precursor, resulting in g-C_3_N_4_ with an improved dispersion. The influence of varying GO concentrations on the properties of g-C_3_N_4_ was systematically compared. The doped g-C_3_N_4_ was integrated into the GO membranes, creating a two-dimensional heterostructure with interconnected channels. These composite membranes exhibited stability in both acidic and alkaline environments over an extended period. They were then applied for the separation of mono- and divalent ions, evaluating their performance in two distinct processes: chemical potential-driven permeation without external pressure and pressure-driven filtration. The GO/g-C_3_N_4_ composite membranes demonstrated superior water permeability and selectivity compared with the GO membranes alone. Furthermore, the membranes were surface-modified using the cationic hydrophilic polymer polyethylenimine (PEI) to enhance their surface charge and hydrophilicity. By adjusting the PEI concentration and the number of coating layers, the membrane’s performance in nanofiltration and permeation was significantly enhanced compared to pure GO membranes, effectively overcoming the typical trade-off between permeability and selectivity.

## 2 Materials and Methods

### 2.1 Materials

Monolayer graphene oxide dispersion (5 mg/g) was purchased from Hangzhou Gaoxi Technology Co. Melamine was purchased from Sinopharm Chemical Reagent Co. Polyacrylonitrile (PAN) ultrafiltration membrane (pore size of 0.22 μm) was purchased from Tai’an Lanjing Trading Co. Potassium chloride, lithium chloride, and magnesium chloride were purchased from Shanghai Macklin Biochemical Technology Co. Polyethylenimine (PEI, molecular weight 70 000 Da) was purchased from Shanghai Aladdin Biochemical Technology Co. The deionized water used in the experiments was produced by an ultrapure water apparatus (GZY-P20-W). All chemicals were used as received without further treatment.

### 2.2 Preparation of Modified g-C_3_N_4_ Nanosheets

Five-gram portion of melamine was introduced into a 30 mL crucible containing 10 mL of deionized water and thoroughly mixed. Subsequently, a specific quantity of GO was incorporated into this mixture and homogenized. The mixture was then subjected to drying in an oven at a temperature of 80 °C for a duration of 12 h. After drying, the solid residue was removed, ground to a uniform consistency, and returned to the crucible. This crucible was subsequently placed in a muffle furnace for calcination at a temperature of 550 °C for a period of 4 h, with a controlled heating rate of 2.2 °C per minute. Once the material had cooled to room temperature, blue-gray g-C_3_N_4_ samples were collected for subsequent applications. These samples were then dispersed in deionized water at predetermined ratios and sonicated continuously for 2 h to form modified g-C_3_N_4_ sheets. The ratios of melamine to graphene oxide were varied as 500:1, 200:1, 100:1, and 50:1, with the resulting g-C_3_N_4_ sheets designated as C_1_, C_2_, C_3_, and C_4_, respectively. For comparison, unmodified g-C_3_N_4_ was prepared by following the same method but without the addition of GO.

### 2.3 Preparation of Different Membranes

GO membrane: 1 mg of GO was dispersed in 50 mL of deionized water and subjected to ultrasonication for 5 min to achieve a homogeneous suspension. A membrane was then formed on a polyacrylonitrile (PAN) substrate by using the vacuum filtration method. The resulting membrane was first dried in an oven at 45 °C for 1 h, followed by a secondary drying step at 80 °C for another hour.

GO/g-C_3_N_4_ membrane: 0.5 mg of graphene oxide and 0.5 mg of modified g-C_3_N_4_ were dispersed in 50 mL of deionized water, and the other steps were the same as those described above for the preparation of the GO membrane. According to the modified g-C_3_N_4_ used, this composite membrane was named as C_1_GO membrane, C_2_GO membrane, C_3_GO membrane, and C_4_GO membrane.

**Scheme 1:**
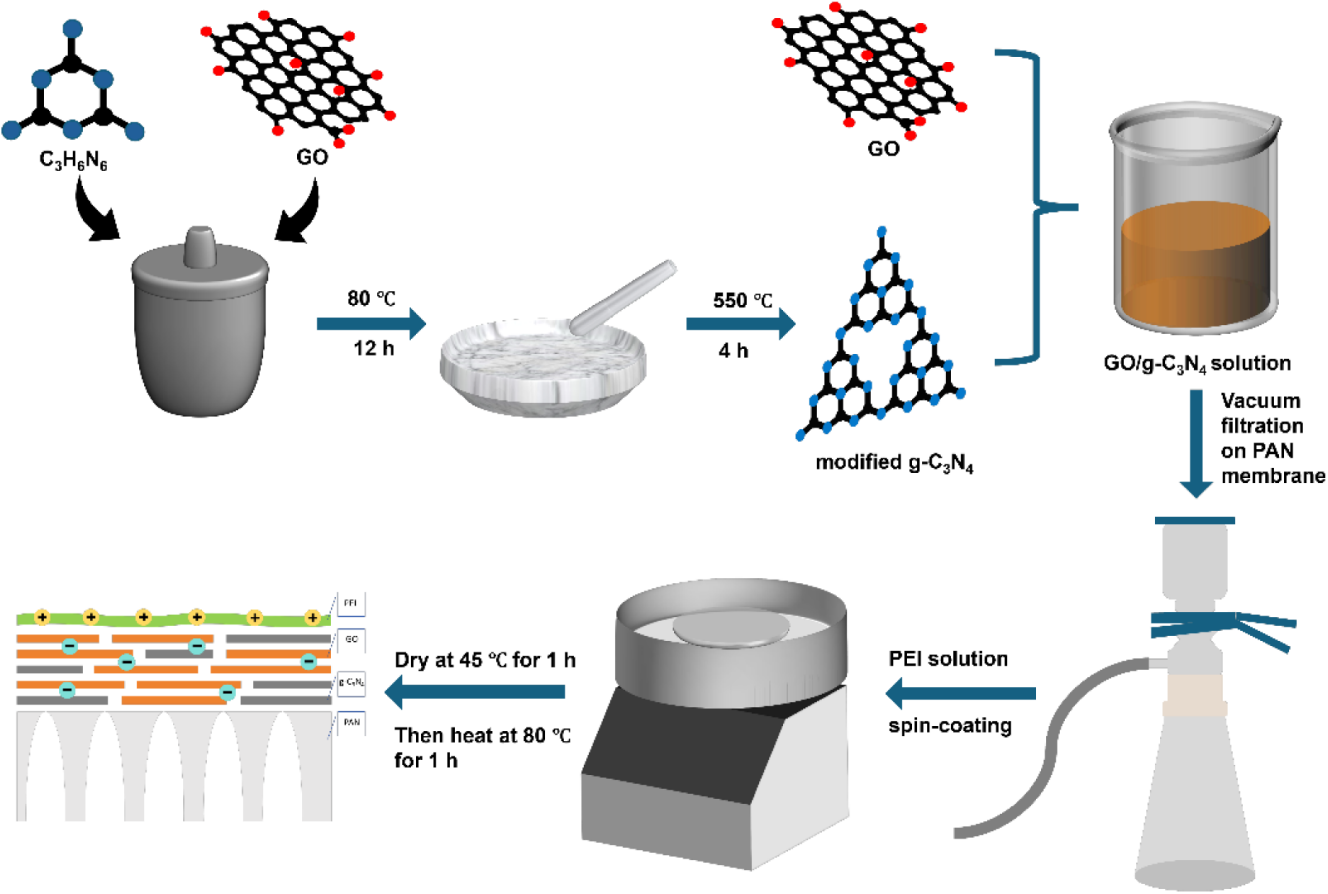
Schematic Illustration of the Fabrication Procedure of the PEI-Modified GO/g-C_3_N_4_ Membrane

PEI-modified GO/g-C_3_N_4_ membrane: The GO/g-C_3_N_4_ membranes, after filtration, were mounted on a Spin Coater (model KW-A4). Solutions of polyethylenimine (PEI) with varying mass fractions of ω = 0.01, 0.03, 0.05, 0.07, and 0.1 were applied to the membrane surfaces via spin coating, as shown in Scheme 1. The number of coating layers was set at 1, 3, 5, and 7. Following the spin coating, the membranes were dried in an oven at 45 °C for 1 h. Subsequently, they were heat-treated in an oven at 80 °C for another 1 h to yield the PEI-modified GO/g-C_3_N_4_ membranes. The naming convention for these membranes was PEI*x* – *y*/CGO, where “*x*” denotes the mass fraction of PEI and “*y*” indicates the number of layers applied.

### 2.4 Characterization Techniques

Field-emission scanning electron microscopy (FESEM, Hitachi SU8010) was used to characterize the morphology of the surfaces and cross section of the membranes. Several different regions were scanned for each sample, and representative images were selected among them because energy-dispersive X-ray spectroscopy (EDS) was used to characterize the elemental distribution and content of the samples in the selected region. Atomic force microscopy (AFM, Bruker Multimode 8) was used to measure the membrane surface morphology and surface roughness. A contact angle meter (DSA, Kruss DSA30S) was used to detect the static water contact angle to evaluate the hydrophilicity of the membrane surface. X-ray photoelectron spectroscopy (XPS, Thermo Scientific K-Alpha) was used to characterize the elemental composition and content, as well as the chemical structure of the membrane surfaces. X-ray diffraction (XRD, SimartLab SE) was performed by using Cu–Kα as the radiation source to determine the material composition and calculate the interlayer spacing of the membrane. A Fourier-transform infrared spectrometer (FTIR, Thermo NICOLET IS50) was used to characterize the molecular structure and chemical bonding composition of the samples, and the data acquisition process was performed in transmission mode over the range of 500–4000 cm^–1^. A laser Particle Sizer (LPS, Malvern MAZ3000) was used to measure the positive and negative properties of charges carried by aqueous samples. Raman spectrometry (HORIBA, LABRAM HR) was used to determine the structure of the material under a 532 nm laser.

### 2.5 Membrane Performance Test Methods

Permeation test: The ion permeation performance of the membrane was tested by using a homemade permeation assembly. The membrane was fixed between the feed side and the permeation side of the test module, and the effective membrane area was 4.15 cm^2^. In the test, 70 mL of deionized water mixed with 70 mL of 0.5 mol·L^–1^ mono or binary salt solution (monosalt solution including KCl, NaCl, LiCl, and MgCl_2_ solution, binary salt solution including KCl/MgCl_2_, NaCl/MgCl_2_, and LiCl/MgCl_2_ mixed solution) was introduced into the permeation side and feed side of the component, and both sides were put into the magnetic rotor for stirring to eliminate concentration difference. The concentration of the salt solution was measured every 10 min using a conductivity meter (SHKY DDS-11A), and the test lasted for 120 min. The permeation rate of single-component salt ions *J*_p_ (mol·m^–2^·h^–1^) was obtained from the permeation-side salt concentration data over time by linear fitting, expressed as eq 1:
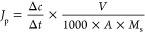
1where Δ*c/*Δ*t* (mg·L^–1^·h^–1^) is the change in ion concentration per unit time of permeation measurement, *V* (L) is the volume of solution measured by permeation, *A* is the effective membrane area (m^2^), and *M*_s_ (g·mol^–1^ is the molecular weight of the salt).

The selectivity of mono/divalent ions was calculated from eq 2:
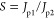
2where *S* is the selectivity of mono/divalent ions, *J*_p1_ is the permeation rate of monovalent ions, and *J*_p2_ is the permeation rate of divalent ions.

The ion separation permeation test of the binary salt solution was performed as described above by injecting the two sides of the feed solution and then taking a certain amount of the permeation test solution for dilution after the permeation process had been carried out for 120 min, and then the diluted sample was tested for the concentration of the ions contained by inductively coupled plasma emission spectroscopy (ICP-OES, PerkinElmer Avio 200) and was calculated with the same eqs 1 and 2 as those described above.

Filtration test: Using the homemade staggered flow filtration equipment, the membrane was fixed in the membrane cell with an effective area of 7.07 cm^2^ and the flow rate was fixed at 150 L·h^–1^, and the membrane to be tested was prepressurized for 5 min at a pressure of 3 bar to perform the pure water flux and salt retention test, in which the pure water flux *J*_f_ (L·m^–2^·h^–1^·bar^–1^) was calculated as in eq 3, and the salt retention rate, *R* (%), was calculated as in eq 4:
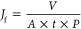
3
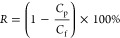
4wherein *V* (L) is the volume of the indicated filtrate, *A* (m^2^) is the effective area of the membrane cell, *t* (h) is the test time, *P* (bar) is the test pressure, and *C*_p_ and *C*_f_ represent the salt concentration of the filtrate and the feed solution, respectively. The concentration is determined in the same manner as that in the above-described permeation test.

## 3 Results and Discussion

### 3.1 Characterization of Modified g-C_3_N_4_ Powder

**Figure 1:**
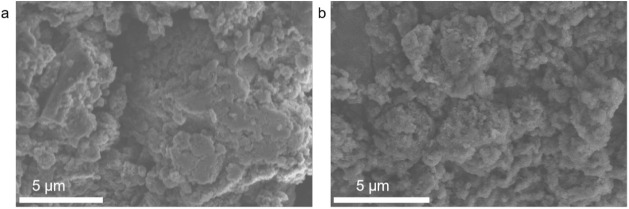
SEM micrograph of (a) g-C_3_N_4_ powder and (b) C_3_ powder.

The morphology of g-C_3_N_4_ powder and GO-doped g-C_3_N_4_ can be observed from the SEM micrograph as shown in Figures 1 and S1. It can be seen that all the samples show the stacking of nanosheets. Compared with the unmodified g-C_3_N_4_, the surface of the modified C_1_, C_2_, C_3_, and C_4_ nanosheets showed a large number of irregular stacks. This could be an irregular mixture produced during the calcination process, where GO influences the agglomeration, curling, and enrichment with melamine at high temperatures, leading to the formation of irregular shapes. Moreover, the color of the samples also changed from light yellow to blue-gray due to the doping of GO. In order to prove the influence of GO on the melamine calcination process, a series of characterization of the samples, such as XPS, FTIR, XRD, and Raman spectroscopy, were carried out.

**Figure 2:**
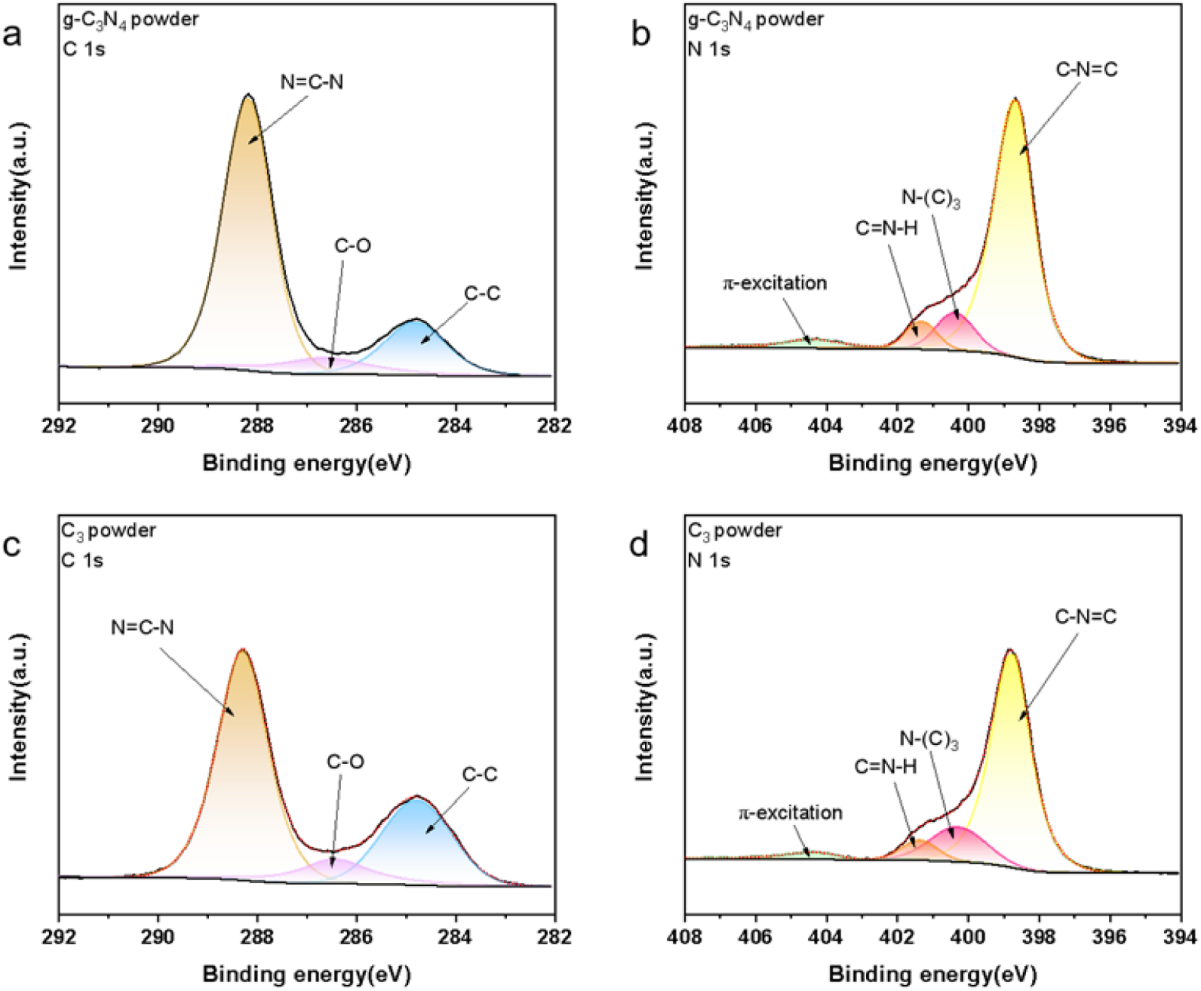
(a) C 1s spectrum and (b) N 1s spectrum of g-C_3_N_4_ powder; (c) C 1s spectrum and (d) N 1s spectrum of C_3_ powder.

The chemical compositions of the powder samples were analyzed by using X-ray photoelectron spectroscopy (XPS), with the resulting spectra depicted in Figures 2 and S2. The C 1s XPS spectra for both pristine g-C_3_N_4_ and modified g-C_3_N_4_ powders exhibited peaks at approximately 284.8, 286.4, and 288.2 eV. These peaks are attributed to the C–C bonds in the triazine ring, C–O bonds, and the sp^2^ hybridized carbon within the N–C=N structure, respectively.^37,38^ The C–C, N=C–N bond is the inherent chemical structure of g-C_3_N_4_, while the C–O bond may originate from oxygen and carbon dioxide present during calcination.^39^ The N 1s XPS spectra were resolved into four distinct peaks with binding energies at 398.7, 400.3, 401.4, and 404.3 eV. These correspond to the sp^2^ hybridized N atoms, the sp^3^ hybridized N atoms, charging effects, and π-excitation on the triazine ring N atoms, respectively.^37,40^ Upon comparison of the XPS spectra for the primary elements present in the materials, it was found that the modified and unmodified samples share identical characteristic peaks, with no distinct peaks indicative of GO materials being detected.

**Figure 3:**
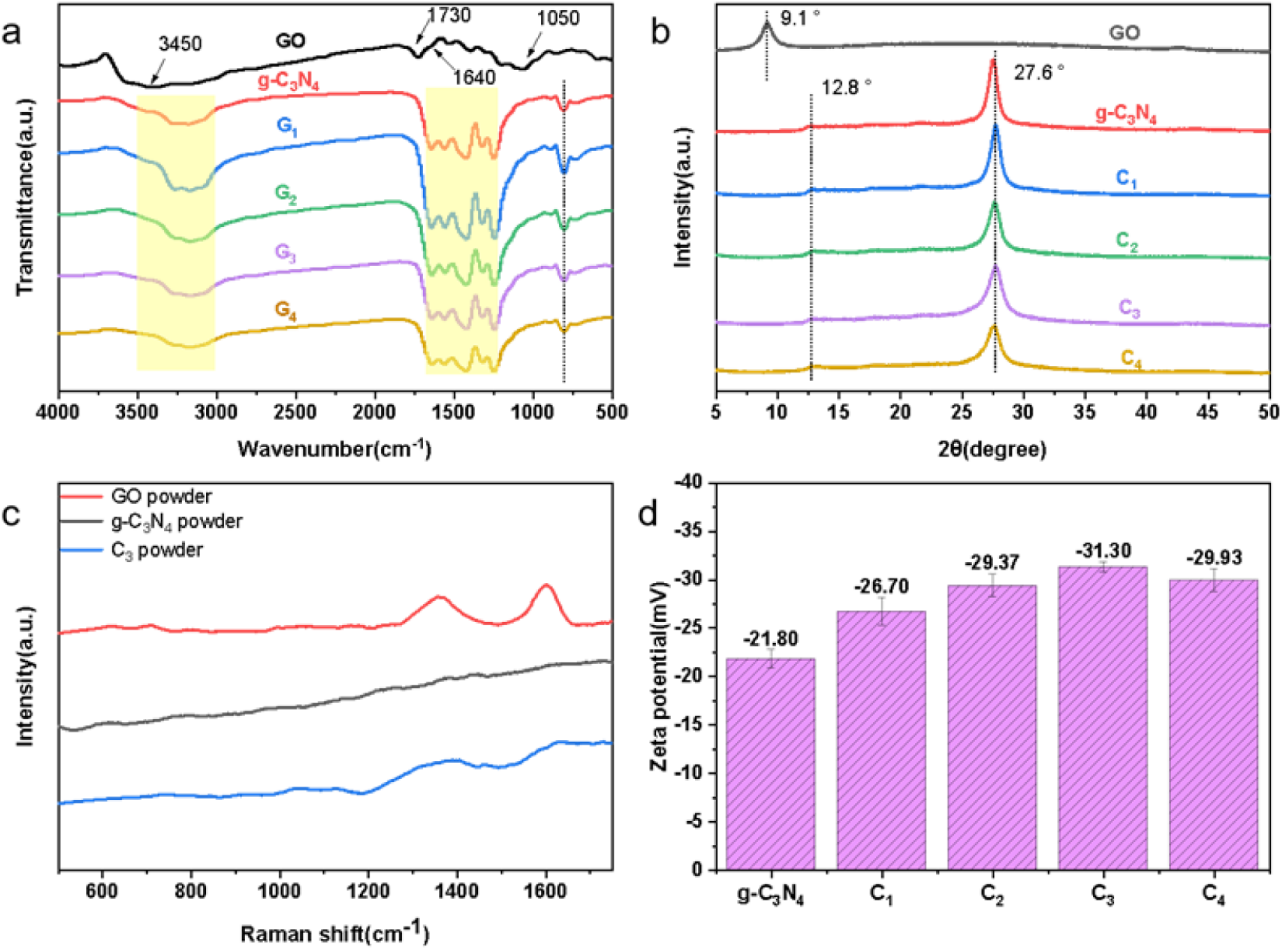
(a) FTIR spectra, (b) XRD spectra, (c) Raman spectra, and (d) zeta potential spectra of GO, g-C_3_N_4_, and modified g-C_3_N_4_ powders.

To further elucidate the chemical composition of the materials, additional analyses were conducted on the synthesized powders. The Fourier-transform infrared spectroscopy (FTIR) spectra presented in Figure 3a revealed characteristic peaks for GO at approximately 1050, 1640, 1730, and 3450 cm^–1^, which are assigned to C–O–C, C=C, C=O, and C–OH stretching vibrations, respectively.^41,42^ Both the pristine g-C_3_N_4_ and the modified samples displayed peaks corresponding to the tri-*s*-triazine ring, C–N heterocyclic, and -NH/NH_2_ groups at 810, 1200–1700, and 3000–3500 cm^–1^, respectively.^43,44^ Notably, no significant peaks indicative of GO were observed in the modified samples. As depicted in Figure 3b, the X-ray diffraction (XRD) pattern of GO showed its (001) crystal plane at 2θ = 9.1°, while both the g-C_3_N_4_ and modified samples exhibited their (100) and (002) crystal planes at 2θ = 12.8° and 2θ = 27.6°, respectively,^45,46^ with no discernible GO characteristic peaks in the modified materials. Furthermore, Raman spectroscopy was employed to characterize the powder samples, as shown in Figure 3c. Under 532-nm laser excitation, the C_3_ samples displayed graphite-like peaks similar to those of GO, suggesting the presence of a small amount of GO on the surfaces of the samples obtained through the calcination of GO-doped melamine. Zeta potential measurements were also performed on the samples’ aqueous solutions at a concentration of 1 mg/mL, as illustrated in Figure 3d. The modified samples exhibited lower negative zeta potential values compared to the unmodified ones, attributed to the presence of oxygen-containing functional groups on the GO. This resulted in a decrease in the negative potential values of the modified samples’ aqueous solutions, aligning with the Raman characterization results indicating the presence of GO on the modified samples’ surfaces. The decrease in potential was correlated to an increase in the amount of GO present.

In summary, the incorporation of GO into g-C_3_N_4_ did not result in significant changes in the chemical structure. However, it did lead to the attachment of a minimal amount of GO to the surface of modified g-C_3_N_4_. This modification altered the color and hydrophilicity of the samples, as shown in Figure S3, and enhanced their dispersion in water. The preservation of the intrinsic structural properties, coupled with the increased specific surface area and improved hydrophilicity, played a pivotal role in enhancing the performance of the resulting membranes.

### 3.2 Characterization of the GO/g-C_3_N_4_ Composite Membrane

**Figure 4:**
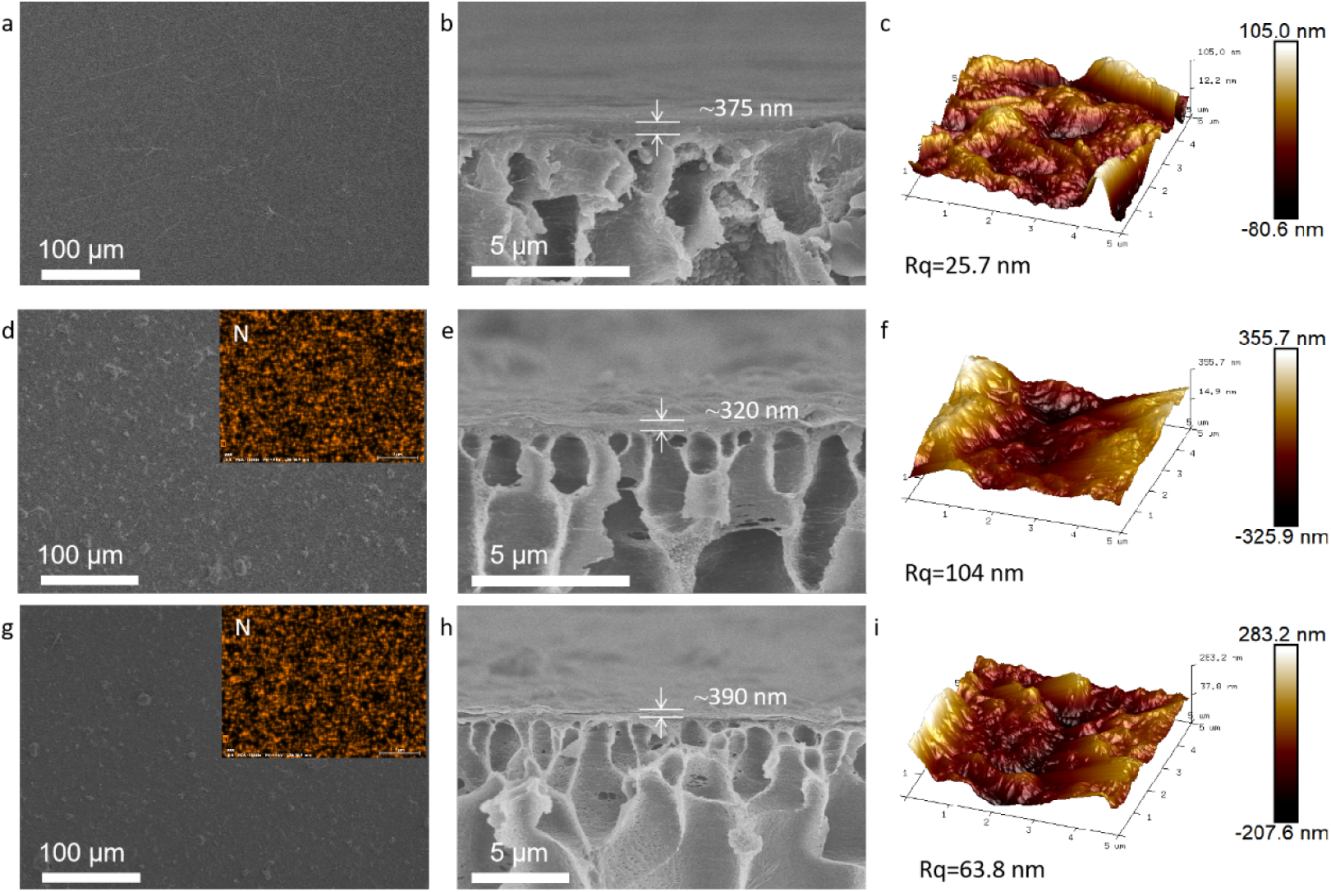
(a) The surface SEM micrograph, (b) the cross-sectional SEM micrograph, and (c) AFM image of the GO membrane; (d) the surface SEM micrograph, (e) the cross-sectional SEM micrograph, and (f) AFM image of C_3_GO membrane; (g) the surface SEM micrograph, (h) the cross-sectional SEM micrograph, and (i) AFM image of PEI_0.07_-5/C_3_GO membrane.

Figure 4a–c illustrates the morphological characteristics of the GO membranes, which align with previous research, indicating that these membranes possess a smooth surface with occasional folds and no significant defects.^47^ The composite membrane incorporating modified g-C_3_N_4_ exhibits a wrinkled surface with the presence of some irregular nanosheets, a feature that contributes to increased surface roughness and a larger specific surface area due to disordered stacking. Figure 4d–f and g–i depicts the C_3_GO film and the PEI_0.07_-5/C_3_GO membrane that has been coated with PEI, respectively. The energy-dispersive X-ray spectroscopy (EDS) surface analysis detects the presence of elemental nitrogen, which can be attributed to the incorporation of g-C_3_N_4_. The cross-sectional view of the membranes reveals that the thickness of the three membranes is similar, suggesting that their properties are likely to be comparable. Furthermore, the AFM data presented in Figure 4 indicate that the incorporation of g-C_3_N_4_, which possesses greater roughness, leads to a substantial increase in the surface roughness of the membranes when compared with the pristine GO membranes. Notably, the surface roughness of the PEI_0.07_-5/C_3_GO membranes is reduced compared to the C_3_GO membranes. This reduction could be due to the polymer coating improving the membrane surface, as PEI not only fills in the physical defects on the membrane surface but also enhances the hydrophilicity of the membranes by its coating.^48^

**Figure 5:**
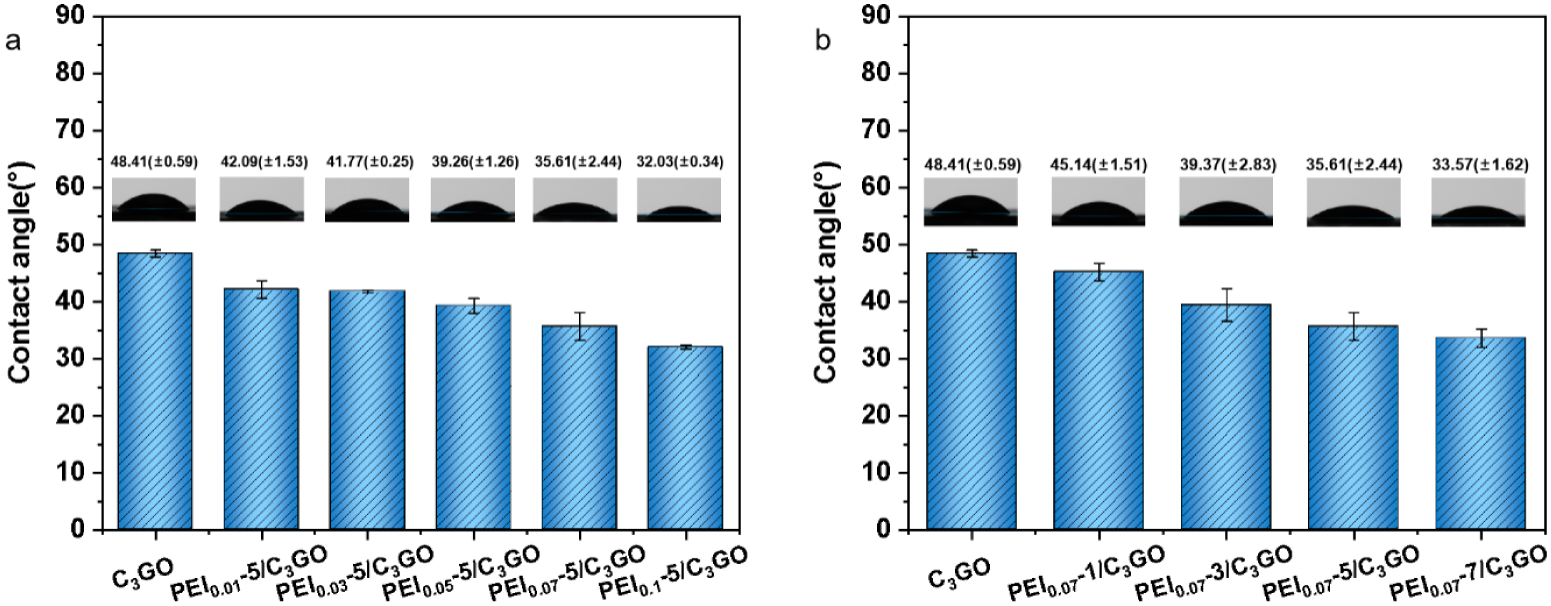
Water contact angle of composite membranes (a) coated with different PEI concentrations and (b) with different numbers of PEI-coated layers.

The PEI’s presence on the membrane surface imparts a stronger hydrophilic character to the material. Consequently, we conducted measurements of the contact angle for membranes coated with varying concentrations and layers of PEI. Figure 5 shows that the water contact angle on the membrane surface decreases progressively with increasing PEI concentration and the number of coating layers, which signifies an enhancement in the hydrophilicity of the membrane surface. The instability of GO membranes in water is a well-documented issue due to their flexible structural nature, while the rigid structure of g-C_3_N_4_ poses challenges in processing and membrane fabrication.

To address this, we combined the two materials to create a composite membrane that leverages the benefits of both. We assessed the stability of the GO, C_3_GO, and PEI_0.07_-5/C_3_GO membranes in aqueous environments with pH values of 2, 7, and 14 over time. It was observed that the GO membrane structure began to separate and fragment in neutral and alkaline conditions (pH = 7 and 14). This is attributed to the enhanced deprotonation ability of the oxygen-containing groups in GO under alkaline conditions, leading to an increase in edge charge and hydrophilicity of the GO nanosheets, which in turn increases the interlayer instability and results in the destruction of the membrane structure.^49^ In contrast, the C_3_GO and PEI_0.07_-5/C_3_GO membranes maintained their structural integrity across all three pH environments. The ″rigid-flexible″ nanosheet overlap layer in the composite membrane contributes to its enhanced structural stability.^50^ Additionally, the reduced amount of GO in the composite membranes minimizes the deprotonation effect, allowing the composite membranes to maintain their structural stability (Figures S4–S6).^51^

**Figure 6:**
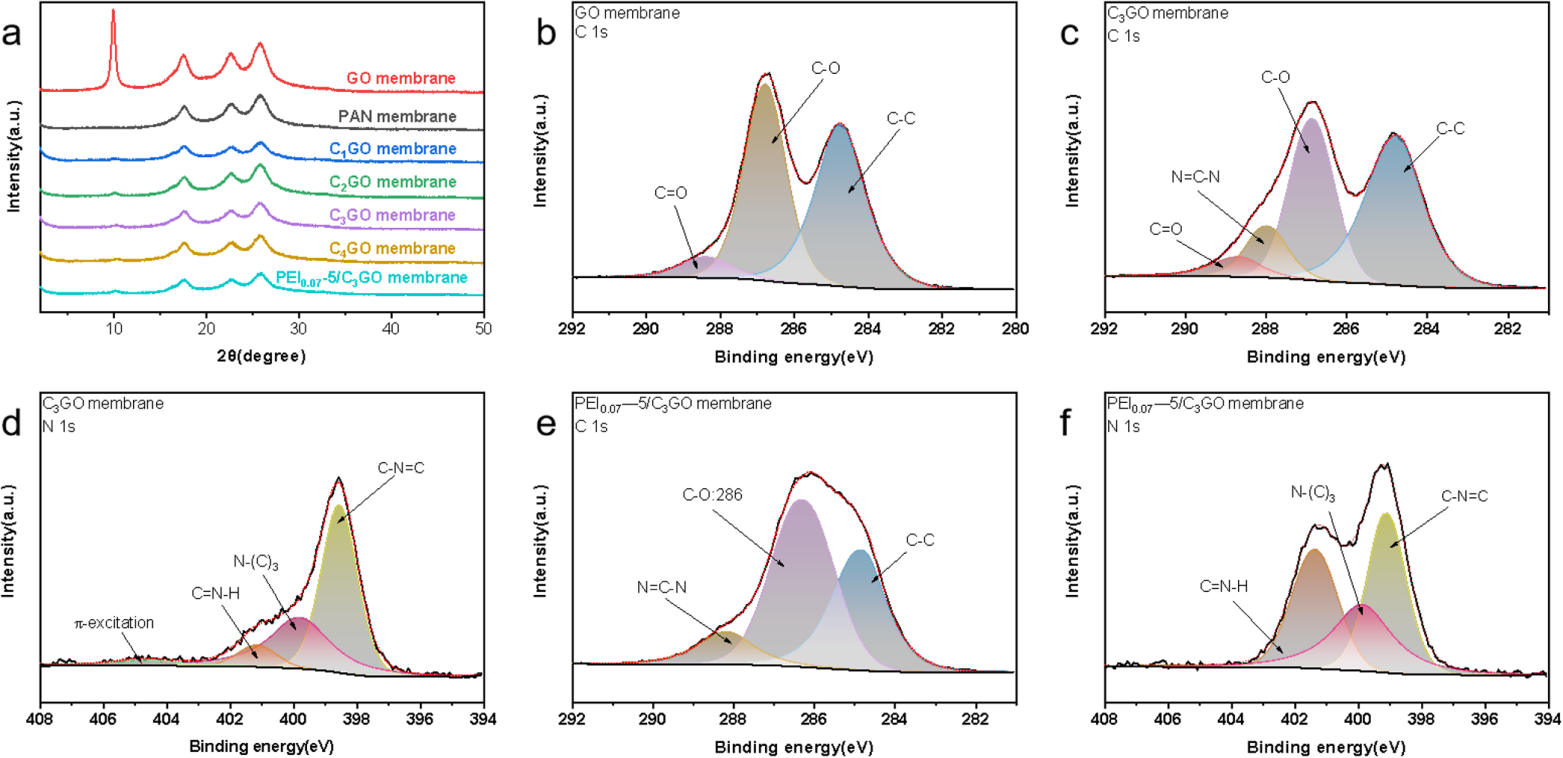
(a) XRD spectra of the membranes. (b) C 1s spectra of the GO membranes. (c) C 1s and (d) N 1s spectra of the C_3_GO membranes. (e) C 1s and (f) N 1s spectra of the PEI_0.07_-5/C_3_GO membranes.

As shown in Figure 6a, the XRD analysis of the membranes reveals the positions of the characteristic peaks for the GO (001) crystalline surface, which are located at 2θ = 9.92°, 2θ = 9.98°, 2θ = 10.22°, 2θ = 10.22°, 2θ = 10.24°, and 2θ = 10.28°. According to Bragg’s law,^52^ the spacing between the nanosheets in the pure GO membranes is reduced compared to the GO powder samples. This reduction may be attributed to the heat treatment during membrane preparation, which likely removes some oxygen-containing groups from the nanosheets, thereby decreasing the layer spacing. In the composite membranes, the characteristic peaks of the GO nanosheets shift to the right, indicating a reduction in the crystalline spacing due to interactions with g-C_3_N_4_. This reduction may enhance the spatial site-barrier effect of the membranes.^53^ In contrast, the interlayer spacing of the PEI_0.07_-5/C_3_GO membrane remains largely unchanged compared to the C_3_GO membrane, suggesting that the surface spin coating does not significantly affect the membrane’s interlayer structure. Figure 6b–f presents the XPS analysis of the membrane surfaces. Compared to the pure GO membrane, the C 1s spectrum of the C_3_GO membrane shows a fitting peak at approximately 288.1 eV,^38^ corresponding to the N=C–N bond in g-C_3_N_4_, as well as a C=O fitting peak at around 288.6 eV attributed to GO.^5^ The absence of the C=O characteristic peak in the C 1s spectrum of the PEI_0.07_-5/C_3_GO membrane suggests a reduction in oxygen-containing functional groups on the membrane surface due to the binding of PEI to GO. This is further supported by a notable enhancement of the C=N–H characteristic peak in the N 1s spectrum, indicating successful PEI binding.

### 3.3 Performance of the Composite Membrane

**Figure 7:**
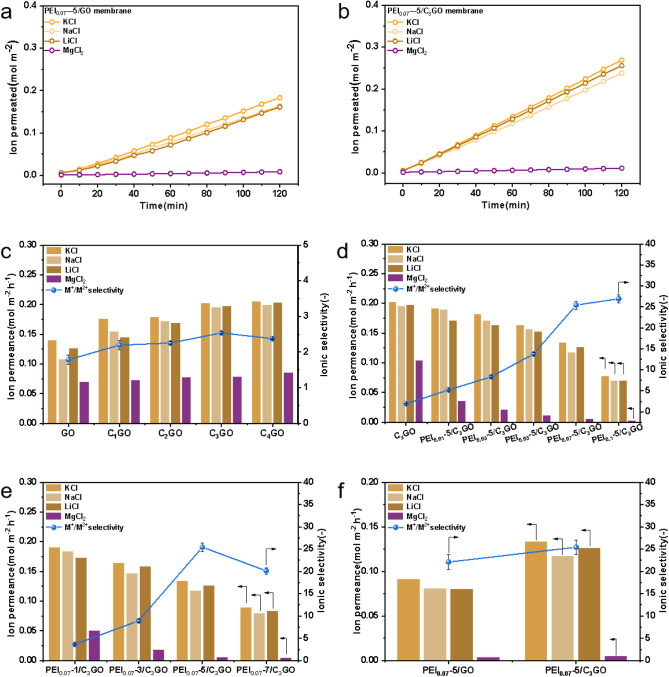
(a) Plot of ion-permeability properties of PEI/GO membranes; (b) plot of ion-permeability properties of PEI_0.07_-5/C_3_GO membranes; comparison of ion permeability and selectivity of composite membranes (c) with different modifications of g-C_3_N_4_ doping, (d) with different PEI concentrations of coating, (e) with different number of PEI coating layers, and (f) PEI-modified GO membrane and PEI-modified C_3_GO membrane.

Permeability testing: To evaluate the ion separation capabilities of the membranes, we employed both permeation and filtration techniques. The permeation experiments were carried out using a custom-built permeation setup (as shown in Figure S7) to test single-component salt solutions with K^+^, Na^+^, Li^+^, and Mg^2+^, as well as mixed salt solutions containing K^+^/Mg^2+^, Na^+^/Mg^2+^, and Li^+^/Mg^2+^. For the single-component solution tests of the composite membranes, we assessed the ion permeation and separation performance of GO composite membranes with varying amounts of modified g-C_3_N_4_ and membranes with different PEI concentrations and coating layers. The results, as depicted in Figures 7a,b and S8–S10, indicated that the ion concentration generally increased linearly with time, and the permeation rates followed the order K^+^ > Na^+^ > Li^+^ > Mg^2+^. This trend corresponds to the hydrated ionic radii (K^+^ (3.31 Å) < Na^+^ (3.58 Å) < Li^+^ (3.82 Å) < Mg^2+^ (4.28 Å)),^31,54^ consistent with previous research suggesting that hydrated ions must shed all or part of their hydration layer to pass through the membrane. Ions with the same charge exhibit different affinities for the hydration layer due to differences in ionic radii.^54,55^ The selective passage of ions through the membrane is attributed to variations in permeation rates arising from size exclusion and differences in the energy required for dehydration.

In this study, the composite membranes incorporating modified g-C_3_N_4_ exhibited a markedly improved monovalent ion permeation rate compared to the pristine GO membranes (Figure 7a–c). The introduction of g-C_3_N_4_ led to the formation of GO/g-C_3_N_4_ nanofluidic heterostructure channels within the composite membranes, which were randomly distributed. These channels, along with the inherent voids present in the g-C_3_N_4_ planes, provided additional pathways for water molecules, reduced mass transfer resistance, and facilitated faster water transport, resulting in the observed enhancement of permeability due to the ultralow friction water flow. Furthermore, the presence of some closely spaced g-C_3_N_4_/g-C_3_N_4_ channels within the membrane layer posed a significant barrier to the passage of larger divalent ions, thereby improving the selectivity between monovalent and divalent ions in the composite membranes. Among the membranes tested, the C_3_GO membrane demonstrated the most outstanding performance in terms of permeability and selectivity, showing a permeability increase of approximately 60% over the pure GO membrane and a selectivity 1.43 times greater than that of the pure GO membrane. As a result, the C_3_GO membrane was selected for further investigation.

Based on the C_3_GO membrane, we applied PEI solutions of varying concentrations and layer counts via spin coating to further enhance the membrane’s selectivity for monovalent over divalent ions by leveraging the Donnan effect,^56^ which is amplified by the hydrophilic and positively charged nature of PEI. As depicted in Figure 7d,e, the permeation rates for both monovalent and divalent ions were diminished under the influence of PEI, with the reduction becoming more pronounced as the concentration of PEI and the number of coating layers increased. Notably, the impact on divalent ions was more significant than on monovalent ions, leading to a marked improvement in the composite membrane’s selectivity.

By increasing the PEI concentration, the density of positive charge on the membrane surface was increased. When the PEI solution concentration increased from ω = 0 to ω = 0.1, the permeation rate for monovalent ions dropped from an average of 0.1836 mol·m^–2^·h^– 1^ for the uncoated C_3_GO membranes to 0.0722 mol·m^–2^·h^–1^, and for divalent ions, it decreased from 0.0353 to 0.00268 mol·m^–2^·h^–1^. The PEI solution at ω = 0.07 resulted in monovalent and divalent ion permeation rates of 0.1254 and 0.00493 mol·m^–2^·h^–1^, respectively, which were comparable to the rates achieved with ω = 0.1 but with nearly double the permeability and similar selectivity. Therefore, the PEI solution at ω = 0.07 was selected for further experiments. Similarly, increasing the number of PEI coating layers from 1 to 7 affected the composite membrane’s permeability selectivity due to the increased charge density and the addition of a thicker PEI layer, which posed a more substantial barrier to ion permeation. This barrier led to a decrease in the mono/divalent ion selectivity when the coating reached 7 layers. Consequently, the PEI_0.07_-5/C_3_GO membrane, coated with 5 layers of PEI, was identified as the optimal performing composite membrane in the study. It exhibited monovalent and divalent ion permeation rates of 0.1254 and 0.00493 mol·m^–2^·h^–1^, respectively, and a selectivity of 25.4, representing a 14-fold increase in selectivity compared to the pure GO membrane and surpassing the traditional trade-off limitations. Finally, we used the PEI/GO membrane without g-C_3_N_4_ doping as a control. It can be seen that the composite membrane containing g-C_3_N_4_ has better permeability and selectivity.

**Figure 8:**
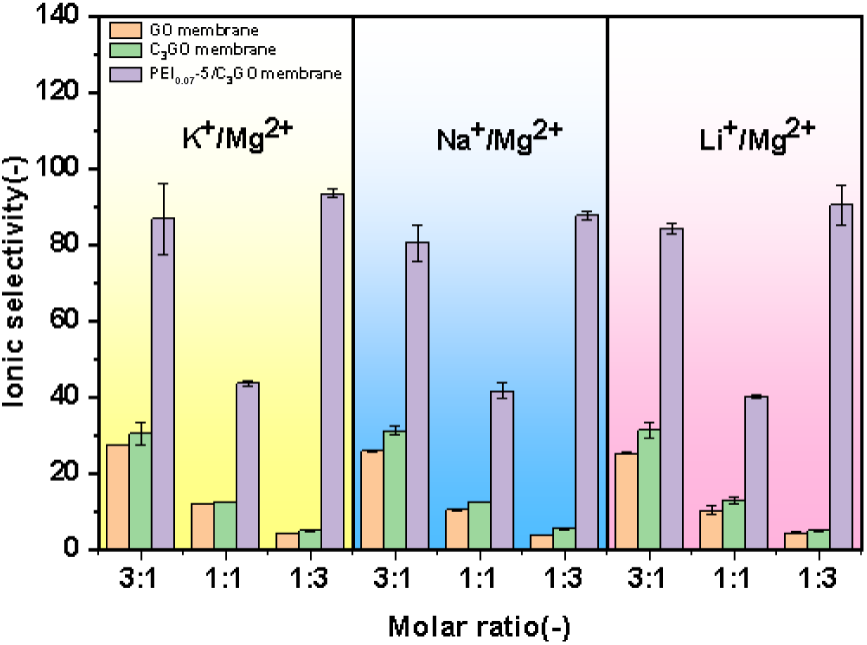
Comparison of ion selectivity of GO, C_3_GO, and PEI_0.07_-5/C_3_GO membranes in different ratios of binary mixed salt solutions.

Furthermore, we conducted ion permeation tests on binary mixed solutions with varying mono- and divalent ion concentration ratios using pure GO membranes, C_3_GO membranes, and PEI_0.07_-5/C_3_GO membranes. As illustrated in Figure 8, for the GO and C_3_GO membranes without PEI surface coating, higher selectivity for monovalent over divalent ions was observed when the molar concentration of monovalent ions exceeded that of divalent ions in the mixture. However, this selectivity was significantly reduced when the molar concentration ratios were equal or when the molar concentration of divalent ions was greater. In contrast, the PEI_0.07_-5/C_3_GO membranes demonstrated superior ion permeability compared to the uncoated C_3_GO membranes across all three concentration ratios tested. They also showed higher selectivity than that of the uncoated PEI membranes, particularly in tests with higher concentrations of divalent ions. This enhanced performance is attributed to the competitive effect of monovalent and divalent ions in binary salt solutions.^57^ Monovalent ions have higher diffusion coefficients than divalent ions, and greater divalent ion concentrations are subjected to greater resistance to operation on the surface of membranes bearing the same charge.^58^ Thus, relative to the single-component mutual comparison, the mono/divalent ion separation ratio in binary salt solutions reached a more significant 90.4.

**Figure 9:**
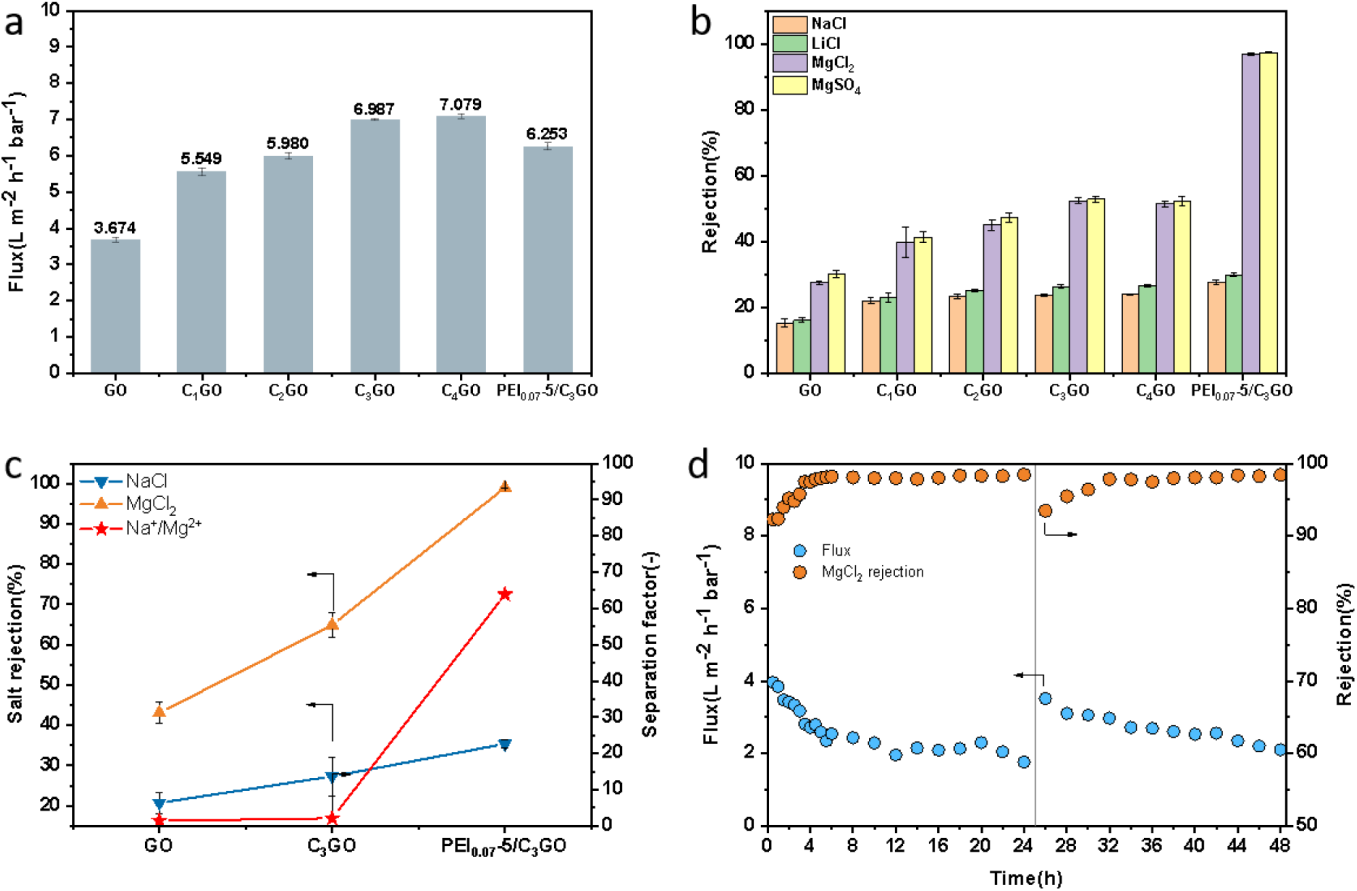
(a) Pure water flux performance, (b) mono- and divalent ion retention performance of GO, C_1_GO, C_2_GO, C_3_GO, C_4_GO, and PEI_0.07_-5/C_3_GO membranes, (c) mono- and divalent ion selectivity comparison plots of GO, C_3_GO, and PEI_0.07_-5/C_3_GO membranes in binary mixed salt solution, and (d) MgSO_4_ solution cycle filtration test performance graph.

Nanofiltration performance: To further test the usability of the composite membranes, we performed nanofiltration performance tests for pure water flux, monosalt, and binary mixed salt solution retention using a homemade staggered-flow filtration apparatus (Figure S11) to demonstrate their desalination potential under external pressure. Figure 9a illustrates the results of the filter test, which indicate that the composite membranes incorporating modified g-C_3_N_4_ exhibit increased water fluxes. This improvement is attributed to the reduced frictional slip within the heterogeneous channels and the presence of a greater number of pores and voids. The permeation rate enhancement is further accentuated by the additional driving force provided by the nanofiltration process. Specifically, the pure water fluxes of the C_3_GO and C_4_GO membranes show an approximate 90% increase compared to those of the pure GO membranes.

In the single salt solution test, nanofiltration was performed with four salt solutions: NaCl, LiCl, MgCl_2_, and MgSO_4_ were conducted at a concentration of 1000 ppm. The results are depicted in Figure 9b. During the single salt retention test, the pure GO membrane exhibited a weak salt rejection capability with low rejection rates for NaCl (15.2%), LiCl (16.1%), MgCl_2_ (27.5%), and MgSO_4_ (30.1%). In contrast, the composite membrane enriched with modified g-C_3_N_4_ demonstrated a superior rejection rate, as previously discussed in the permeation process. The rejection rates for magnesium sulfate were comparatively low, but the composite membrane with modified g-C_3_N_4_ showed enhanced rejection rates, particularly due to the larger size of the ions and the barrier effect within the membrane interlayers under pressure, which made the difference in filtration rates between mono- and divalent ions more pronounced. Consequently, the retention rate for divalent ions increased more significantly than that for monovalent ions. For instance, the C_3_GO membrane showed rejection rates of NaCl (23.8%), LiCl (26.3%), MgCl_2_ (52.2%), and MgSO_4_ (52.8%). The PEI_0.07_-5/C_3_GO membrane, in the nanofiltration test of single-salt solutions, exhibited a notably increased retention of divalent ions to over 95%, while the retention of monovalent ions saw only a minor increase. This indicates that the membrane retains a significant ion screening capability under external pressure.

As illustrated in Figure 9c, during the binary mixed salt solution test, a mixed salt solution of 1000 ppm with a ratio of NaCl to MgCl_2_ of 1:9 was utilized as the test medium, and nanofiltration tests were conducted on the GO membrane, C_3_GO membrane, and PEI_0.07_-5/C_3_GO membrane. In the binary mixed salt solution, the spatial site resistance significantly impacted the passage of divalent ions through the GO and C_3_GO membranes due to interionic competition, leading to a substantial increase in the retention rate of divalent ions compared to the results obtained from the single salt solution tests. Conversely, the PEI_0.07_-5/C_3_GO membrane maintained a high retention rate for divalent ions at 98.9%, with a separation ratio of monovalent to divalent ions of approximately 63.8. Furthermore, we performed a 48 h cycling test on PEI_0.07_-5/C_3_GO composite membranes with 1000 ppm concentration of MgSO_4_ solution in Figure 9d. As the test proceeded, the water flux of the membrane gradually decreased from the initial 3.96 to 1.76 L·m^–2^·h^–1^·bar^–1^ when the test was conducted for 24 h, while the retention rate of MgSO_4_ gradually increased from the initial 92.3% to 98.5%. The water flux of the membrane was reduced by about 50%, so we rinsed the membrane with deionized water for 30 min at this point and then tested it again; the flux of the membrane was recovered after the deionized water cleaning, and the same retention rate was slightly reduced. Therefore, under certain cleaning conditions, the PEI_0.07_-5/C_3_GO composite membrane prepared in this work has a good retention nanofiltration performance.

**Figure 10:**
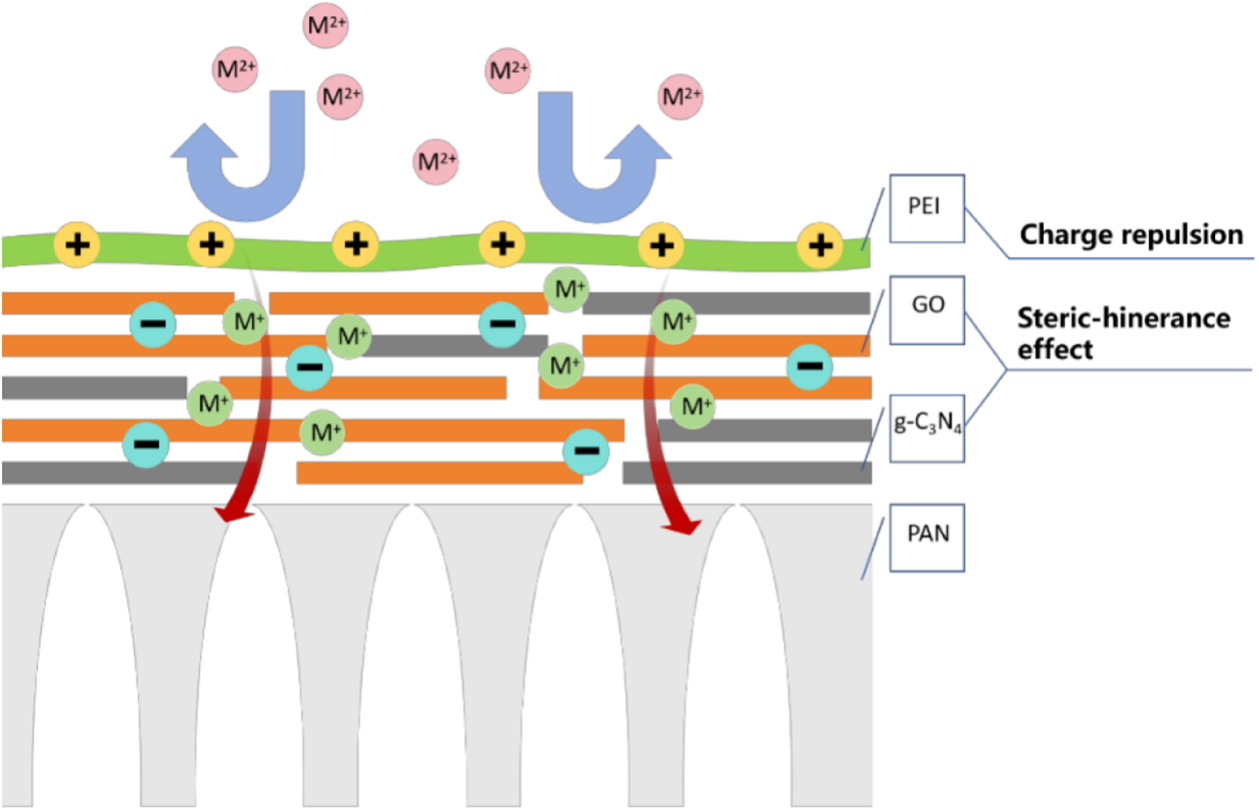
Schematic structure and functional mechanism of PEI/GO composite membrane.

In this work, we designed a PEI_0.07_-5/C_3_GO composite membrane as shown in Figure 10, with a PEI coating over a GO/g-C_3_N_4_ separation membrane on a PAN support body. The ion separation mechanism of the composite membrane is based on charge repulsion with size sieving. In this case, charge repulsion plays a more critical role in the permeation separation process, and the repulsive force of surface charge during permeation without applied pressure resulted in a significant increase in mono/divalent ion selectivity of the PEI-coated composite membrane. During staggered flow filtration with applied pressure, on the one hand, charge repulsion still has an influence on ion separation, while size sieving also plays a key role. Due to the combination of the membrane surface with PEI when the composite membrane surface is coated with PEI makes the oxygen-containing groups on the membrane surface reduced, in addition to the g-C_3_N_4_ intercalation to obtain heterogeneous filtration channels, these structural changes make the divalent ions with larger size subjected to higher mass-transfer resistance, which manifests the effect of sieving under the effect of the applied pressure.

## 4 Conclusions

In this study, graphene oxide (GO) was employed as a modifier to enhance the surface characteristics of graphitic carbon nitride (g-C_3_N_4_). This modification preserved the inherent chemical structure of g-C_3_N_4_ while expanding its surface area and hydrophilicity. Concurrently, by integrating the modified g-C_3_N_4_ into the GO membrane as an intercalation layer, the structural framework of g-C_3_N_4_ augmented the water transport channels within the membrane, which not only significantly improved the permeability and selectivity of the GO membrane but also maximized the iontophoretic flux by approximately 60% for the self-driven membrane and increased the flux by about 90% in the additionally driven nanofiltration test, along with a heightened retention rate for divalent salts. The ″rigid-flexible″ framework structure increased the membrane’s stability as well. Furthermore, modifying the surface charge by applying polyethylenimine (PEI) at specific concentrations and layer numbers enhanced the mono/divalent ion selectivity of the composite membrane. The selectivity of the PEI_0.07_-5/C_3_GO membrane in self-driven mono/divalent ion permeation rose to approximately 25.4, marking a 14-fold increase over that of the pure GO membrane, with selectivity reaching 90.4 in mixed salt solution tests. In nanofiltration with an applied driving force, the retention rate for divalent salts exceeded 95%, and the selectivity remained high at approximately 63.8 in mixed salt solutions.

## Supporting Information Available

SEM micrograph of C_3_GO powders, XPS image of modified g-C_3_N_4_ powders, variation of dispersions of 1 mg/mL g-C_3_N_4_, and modified g-C_3_N_4_ suspensions kept for different periods; comparison of C_3_GO membranes in different pH environments for 14 days comparison of PEI_0.07_-5/C_3_GO membranes in different pH environments for 14 days; ermeability performance of GO and CGO membranes; setup of penetration test; permeation performance of composite membranes coated with different concentrations of PEI; permeation performance of composite membranes coated with different layers of PEI; setup of filtration test equipment (PDF)
