# Supplementary material for: PEI-Reinforced GO/g-C3N4 Composite Membrane for Salt Separation
Source: ACS Omega. 2025 Apr 16;10(16):16620–32. doi: 10.1021/acsomega.5c00135 (PMC12044557; doi:10.1021/acsomega.5c00135)
Supplement: Supplementary file 1 — ao5c00135_si_001.pdf [file ao5c00135_si_001.pdf]

# Supporting information

# for

# PEI-reinforced GO/g-$C_3N_4$ composite membrane for salt separation

*Wenbiao Zheng, Mingfeng Yu, Sujuan Yang, Licong Meng, Yonghe Xiu, Yifan Liu,*

*Hanhui Lei, Terence Xiaoteng Liu*, Zhanhui Yuan*, Liwei Wang**

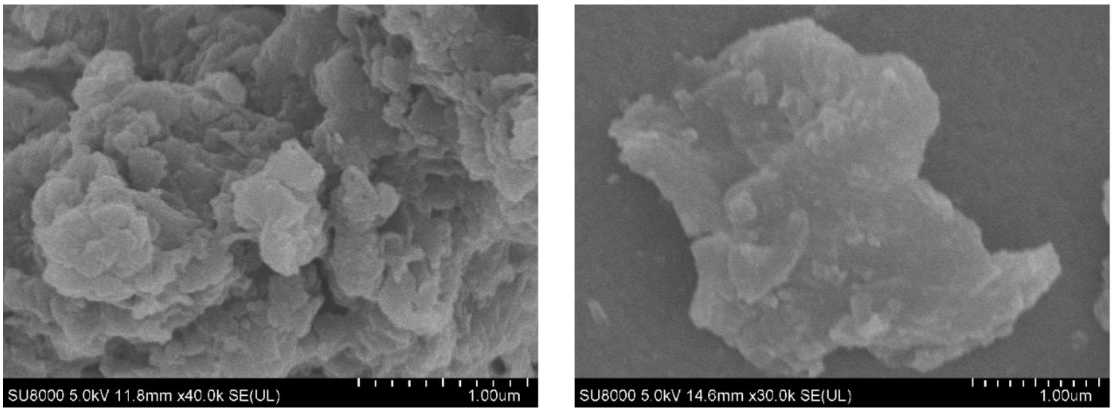


S1. SEM micrograph of $C_3GO$ powders

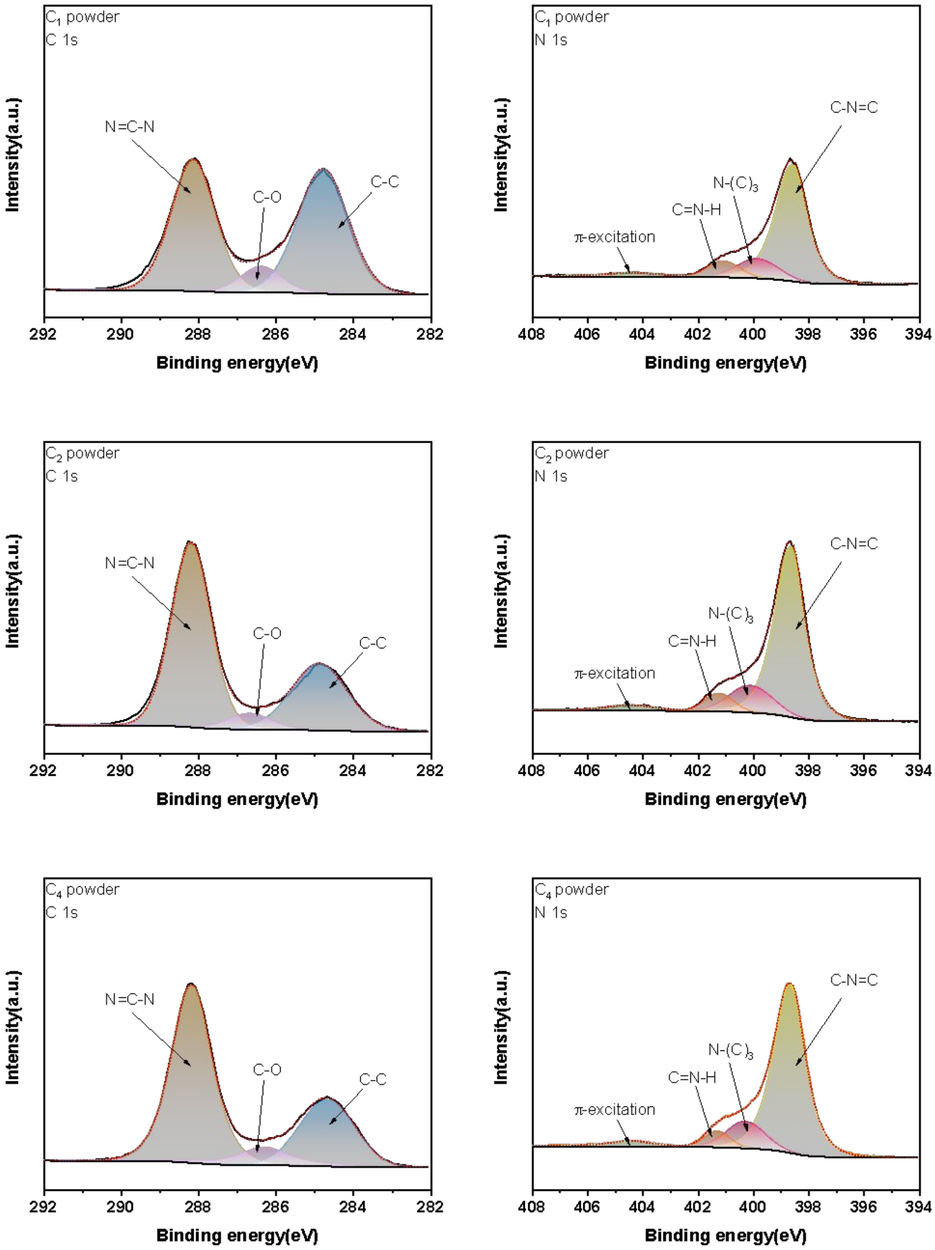


S2. XPS image of modified g-$C_3N_4$ powders

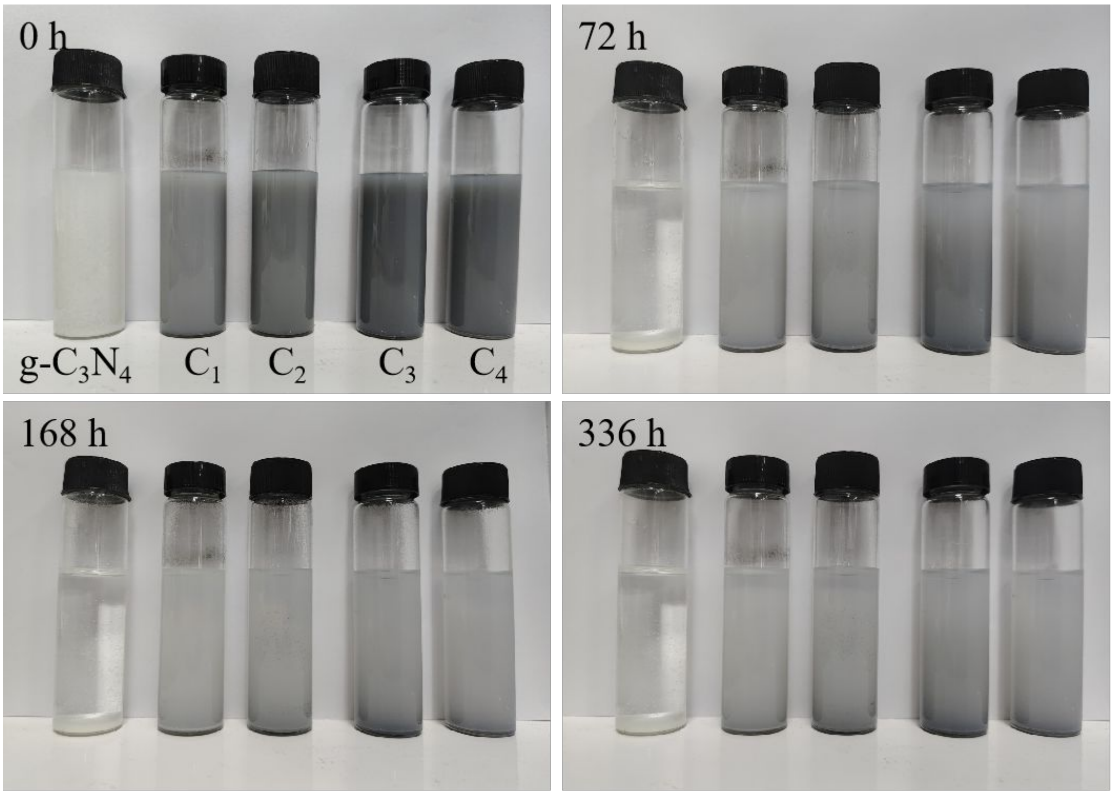


S3. Variation of dispersions of 1 mg/mL g-$C_3N_4$ and modified g-$C_3N_4$ suspensions kept for different periods

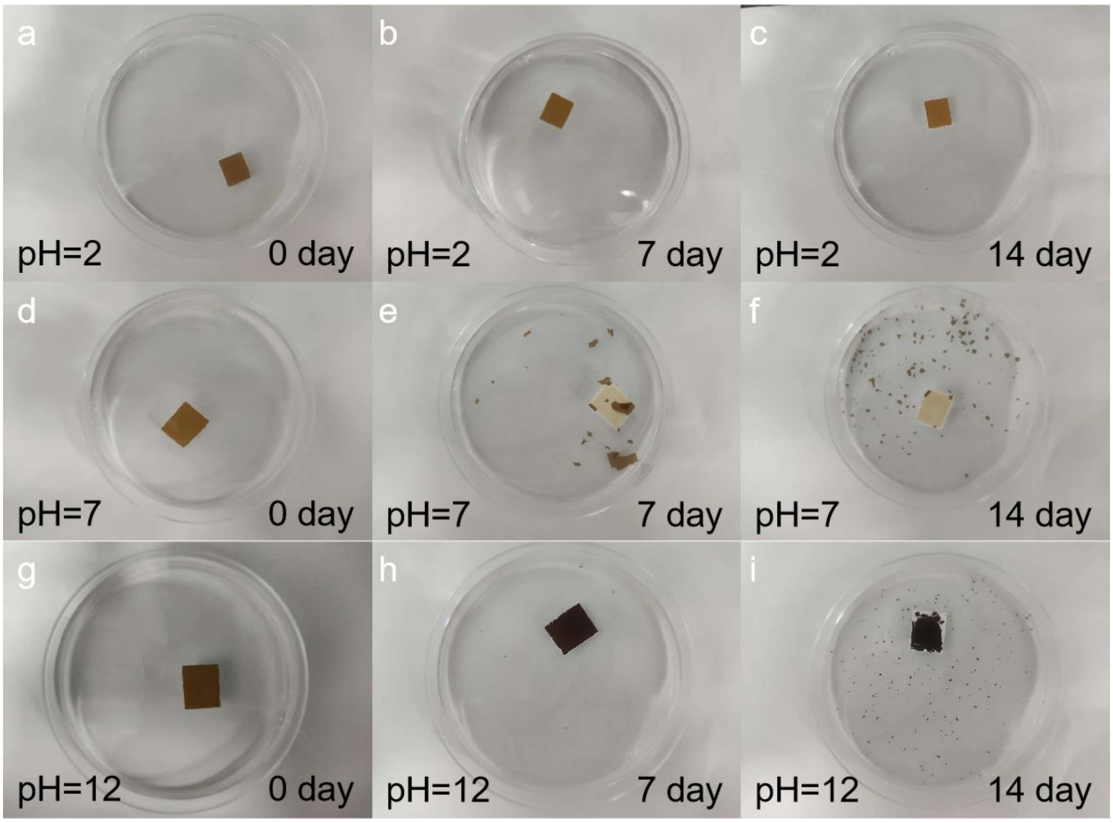

S4. Comparison of GO membranes in different pH environments for 14 days

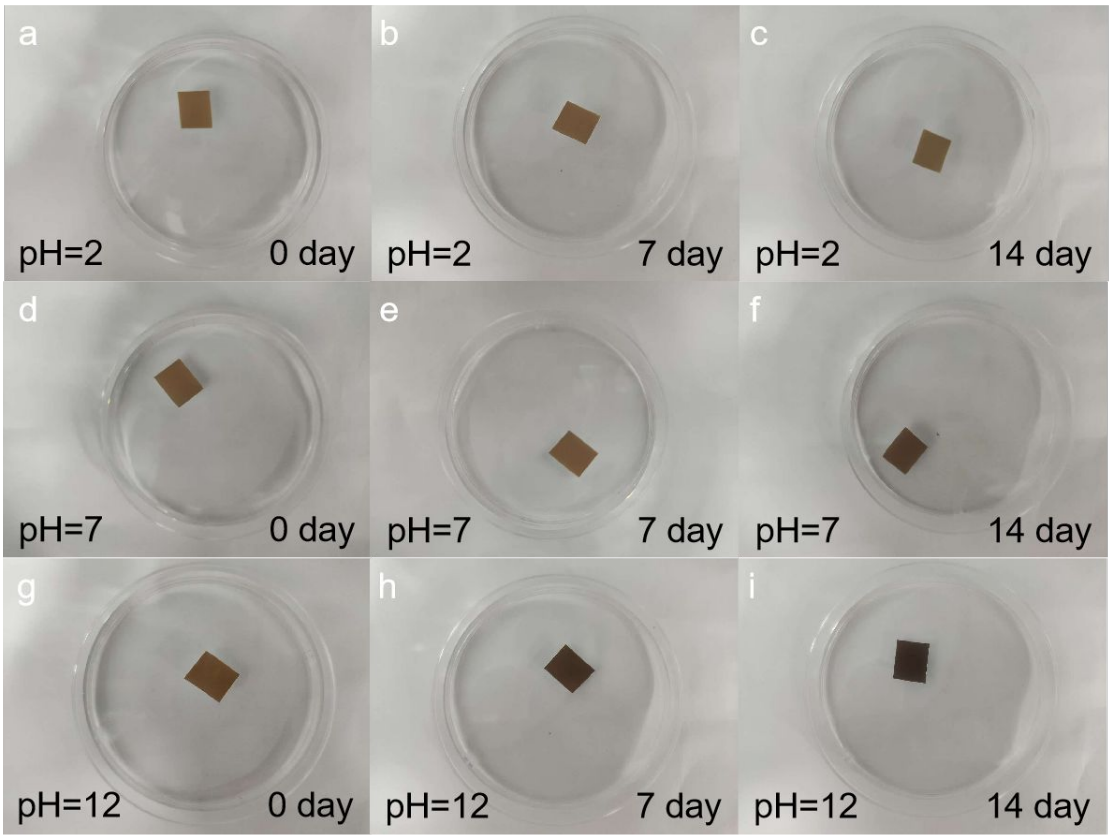


S5. Comparison of $C_3GO$ membranes in different pH environments for 14 days

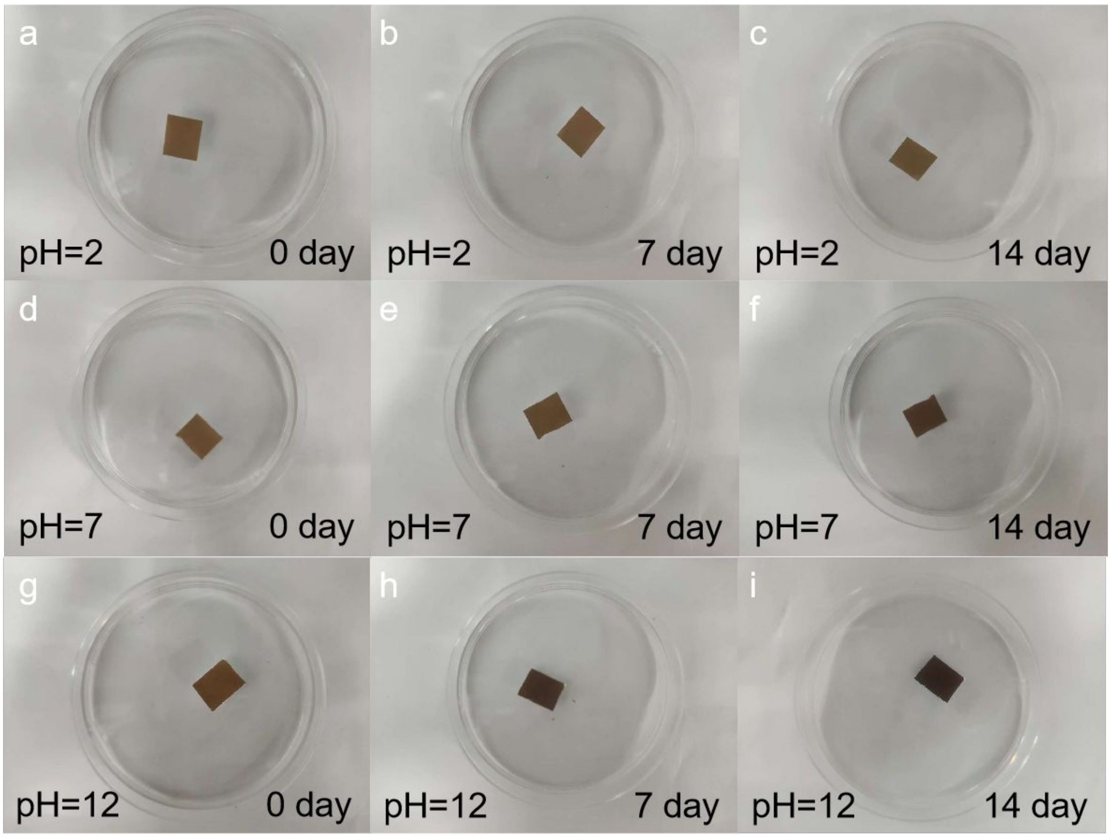


S6. Comparison of $PEI_{0.07}$-5/$C_3GO$ membranes in different pH environments for 14 days

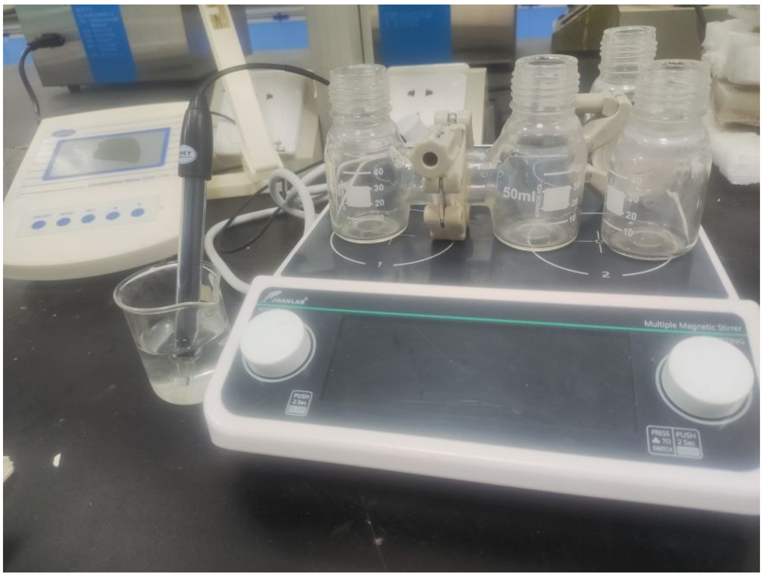


S7. Set-up of penetration test

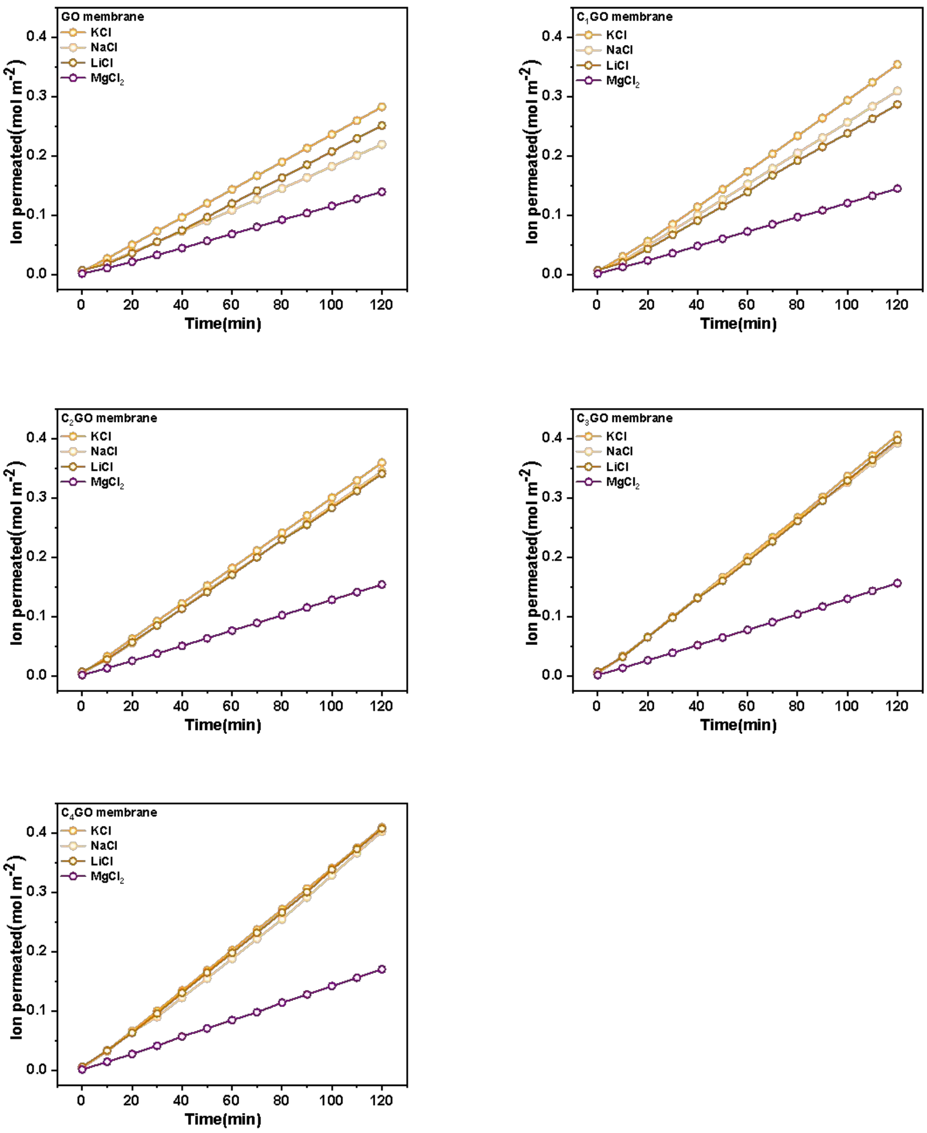


S8. Permeability performance of GO and CGO Membranes

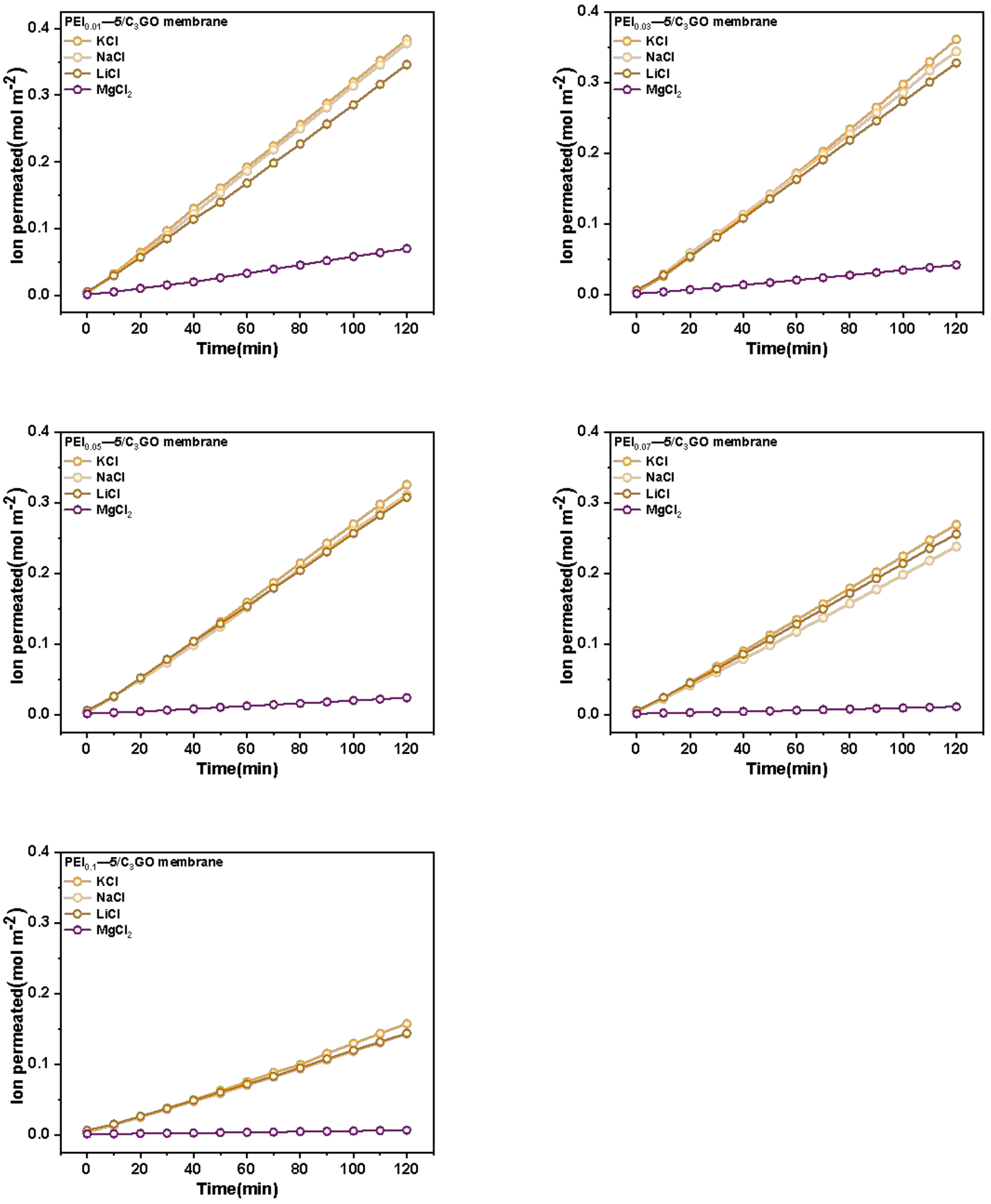


S9. Permeation performance of composite membranes coated with different concentrations of PEI.

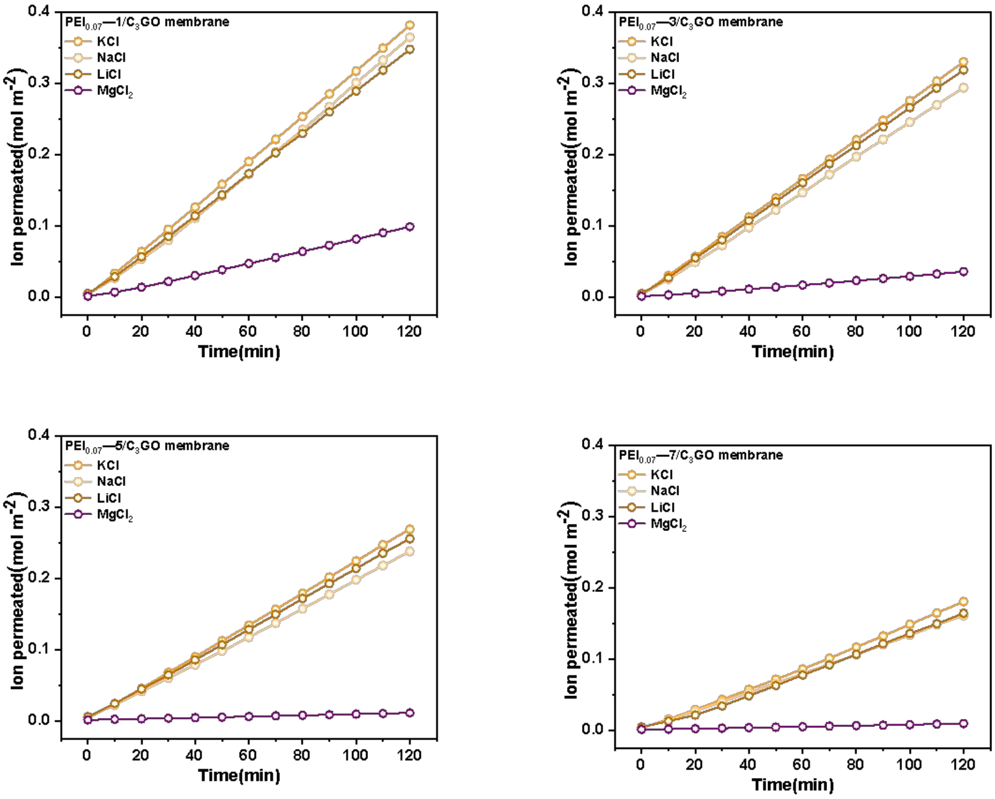


S10. Permeation performance of composite membranes coated with different layers of PEI.

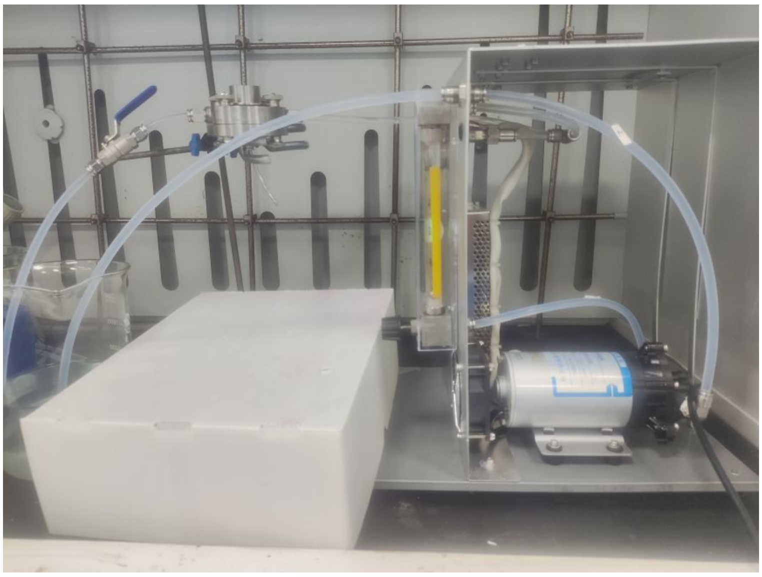

S11. Set-up of Filtration test equipment.
